# Supplementary material for: Rapid Bayesian learning in the mammalian olfactory system
Source: Nat Commun. 2020 Jul 31;11:3845. doi: 10.1038/s41467-020-17490-0 (PMC7395793; doi:10.1038/s41467-020-17490-0)
Supplement: Supplementary file 1 — Supplementary Information [file 41467_2020_17490_MOESM1_ESM.pdf]

Supplementary Information of Rapid Bayesian learning in the mammalian olfactory system

Naoki Hiratani* and Peter E. Latham†

Gatsby Computational Neuroscience Unit, University College London

*N.Hiratani@gmail.com

†pel@gatsby.ucl.ac.uk

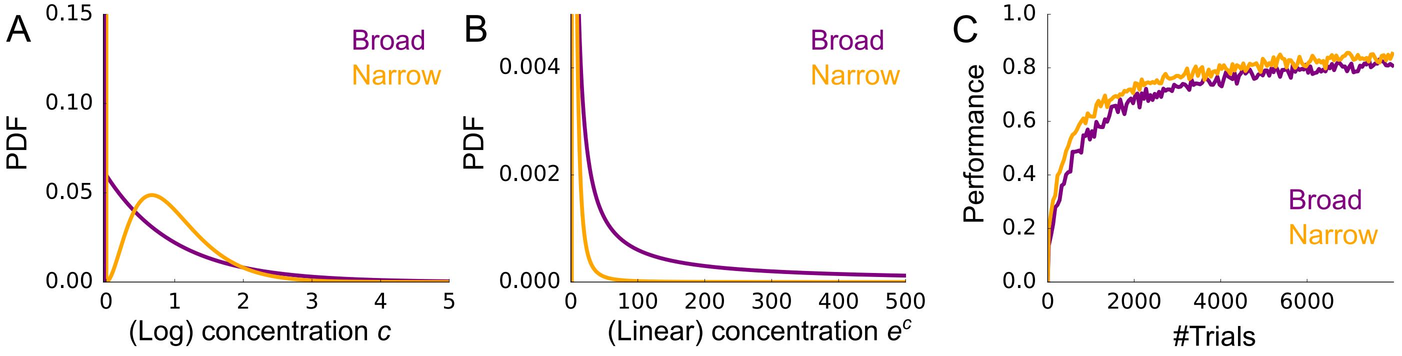

Supplementary Figure 1: **Learning performance under a broader concentration distribution A)** Log-concentration of the odor, c, under the distribution we used in the main text (orange line; slab $\propto c^2 e^{-3c}$), and under a broader distribution (purple; slab $\propto e^{-c}$), the latter chosen to have the same mean as the slab used in the main text. **B)** Same as **A**, but plotted against e^c, which corresponds to the real (linear) concentration, not the log-concentration we used throughout the main text. **C)** Learning performance under the two odor concentration distributions. Performance is only slightly worse under the broader prior.

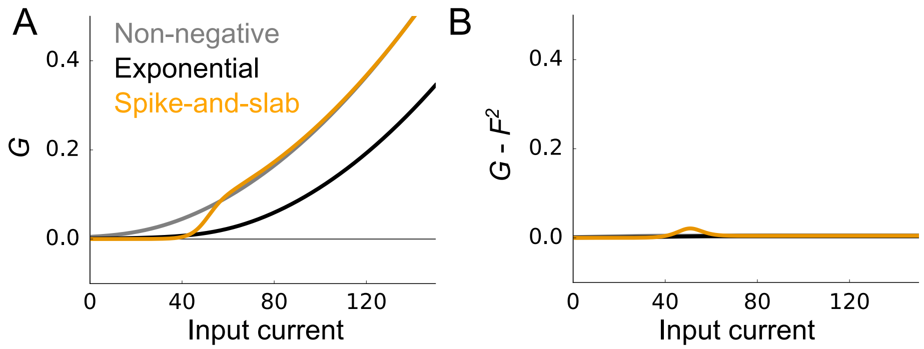

Supplementary Figure 2: **The effect of the prior on the precision update A)** The shape of the transfer function for the precision update $G[y; \bar{c} = 0]$ under the non-negative (gray), exponential (black), and spike-and-slab prior (orange). **B)** The difference between $G[y; \bar{c} = 0]$ and $F[y; \bar{c} = 0]^2$ (ie. [Supp. Fig. 2A] – [Fig. 5A]^2) under the three priors. The gray line for the non-negative prior is hidden under the black line for the exponential prior (they are both nearly zero).
